# Correction: Aging and degradation behaviour of poly(lactic acid) composites in alcoholic and acidic food simulants

**DOI:** 10.1039/d6ra90045c

**Published:** 2026-05-15

**Authors:** Andrei Moldovan, Ionut Sarosi, Violeta Popescu, Stanca Cuc, Codruta Sarosi, Cezara Zagrean-Tuza, Radu Silaghi-Dumitrescu, Ovidiu Nemes, Ancuta-Elena Tiuc

**Affiliations:** a Technical University of Cluj-Napoca, Faculty of Materials and Environmental Engineering 103-105 Muncii Ave Cluj-Napoca Romania ovidiu.nemes@sim.utcluj.ro; b Babeş-Bolyai University, Raluca Ripan Institute for Research in Chemistry 30 Fantanele Street 400294 Cluj-Napoca Romania stanca.boboia@ubbcluj.ro; c Faculty of Chemistry and Chemical Engineering, Babeş-Bolyai University 11 Arany János Street 400028 Cluj-Napoca Romania; d National Institute for Research and Development in Environmental Protection 294 Splaiul Independenţei Blv., District 6 060031 Bucharest Romania

## Abstract

Correction for ‘Aging and degradation behaviour of poly(lactic acid) composites in alcoholic and acidic food simulants’ by Andrei Moldovan *et al.*, *RSC Adv.*, 2026, **16**, 20946–20959, https://doi.org/10.1039/D6RA01374K.

The authors regret two errors introduced into the manuscript.

(1) Firstly, the first sentence in the section titled ‘DSC analysis’ is incorrect. It should read “The samples (Fig. 1) were immersed for six months in food simulants (10% ethanol, 3% acetic acid, and 20% ethanol) and then analyzed by DSC to evaluate changes in thermal behavior (Fig. 11).”

(2) Secondly, the wrong images were incorrectly used in Fig. 10. The correct Fig. 10 is as shown here.

**Fig. 10:**
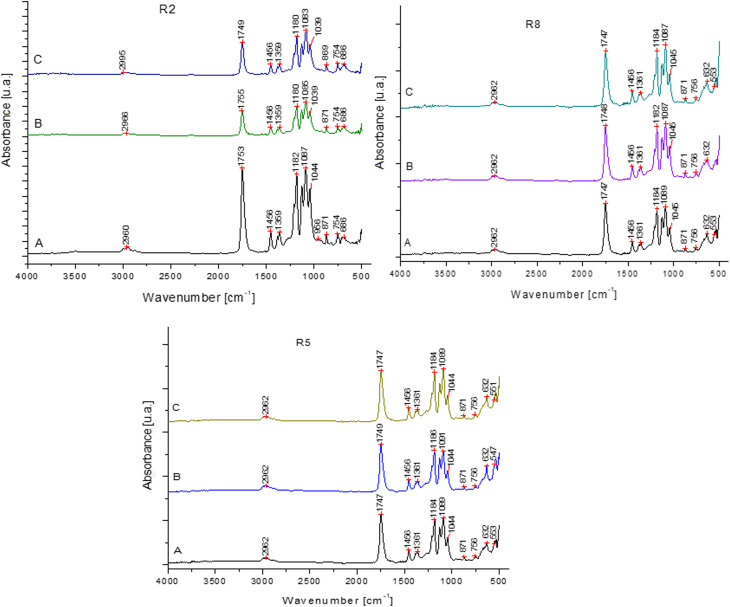
FTIR analysis (initial and after six months of immersion in food simulants).

The Royal Society of Chemistry apologises for these errors and any consequent inconvenience to authors and readers.

